# Perspectives of GPs working in or alongside emergency departments in England: qualitative findings from the GPs and Emergency Departments Study

**DOI:** 10.3399/BJGP.2021.0713

**Published:** 2022-07-26

**Authors:** Helen Anderson, Arabella Scantlebury, Heather Leggett, Chris Salisbury, Jonathan Benger, Joy Adamson

**Affiliations:** York Trials Unit, Department of Health Sciences, University of York, York.; York Trials Unit, Department of Health Sciences, University of York, York.; York Trials Unit, Department of Health Sciences, University of York, York.; Centre for Academic Primary Care, Population Health Sciences, Bristol Medical School, Bristol.; School of Health and Social Wellbeing, University of the West of England, Bristol.; York Trials Unit, Department of Health Sciences, University of York, York.

**Keywords:** emergency department, general practice, general practitioners, qualitative research

## Abstract

**Background:**

Around 43% of emergency department (ED) attendances can be managed in general practice. Strategies to address this include directing appropriate patients to GPs working in or alongside EDs (GPED). Views of GPs choosing to work in GPED roles may inform planning and implementation of GPED services as well as wider general practice provision.

**Aim:**

To explore the experiences and motivations of GPs choosing to work in GPED services in England, and to identify factors that may support or hinder GPs working in GPED roles.

**Design and setting:**

Thematic analysis of 42 semi-structured interviews of GPs working in 10 GPED case sites across England.

**Method:**

Qualitative GP interviews from a mixed-methods study of GPs in GPED roles were thematically analysed in relation to research aims.

**Results:**

Four themes were generated: the ‘pull’ of a portfolio career; the ‘push’ of disillusionment with general practice; professional reciprocity; sustainability of GPED services and core general practice. Flexible, favourable working conditions, collaboration, and professional development made GPED an attractive workplace, often as part of a portfolio career or after retiring from core general practice. Working in GPED services was largely driven by disillusionment with core general practice. Both GPED and core general practice were thought to benefit from GPED GPs’ skills. There were concerns about GPED sustainability and destabilisation of core general practice.

**Conclusion:**

GPED may extend the clinical careers of experienced GPs and support recruitment and retention of more recently qualified GPs. Despite some benefits, GPED may destabilise core general practice and increase pressure on both environments.

## INTRODUCTION

Increased emergency department (ED) attendance continues to place pressure on emergency healthcare systems internationally.^1^^–^^3^

Up to 43% of ED attendances are suitable for management in general practice.^1^ Various strategies to redirect patients who present to ED to GPs have been employed in different countries.^3^ In England, policies have been developed to direct appropriate patients to GPs working in or alongside EDs (GPED)^4^ and this has been supported by capital funding.^5^ In response, several models for co-locating GPs in or alongside EDs have been developed and are set out in a taxonomy.^6^ As well as freeing ED capacity for the sickest patients, such initiatives are expected to improve patient flow and reduce ED crowding, although supporting evidence is limited.^7^

The introduction of GPED services has been undermined by lack of availability and willingness of GPs to work in these settings, leading to gaps in GP rotas.^8^ Simultaneously, general practice more broadly is considered to be at crisis point, with recruitment and retention presenting significant issues both in England^9^^,^^10^ and internationally.^11^^,^^12^ The consequences of role diversification, such as GPED, on general practice more widely (what the authors of the current study term here as ‘core’ general practice, that is, traditional general practice located in local communities) are unclear.

This article explores the experiences and motivations of GPs choosing to work in GPED roles in England, and their views about the role of GPED in relation to core general practice. Findings are used to identify factors that may support or hinder GPs working in EDs and which may be used by policymakers/managers when planning and implementing GPED and general practice provision.

## METHOD

### Design

A large, longitudinal mixed-methods study was carried out to evaluate the impact of GPED on patient care, general practice, acute hospital teams, and the wider urgent care system.^13^ In this paper the authors draw on semi-structured interviews with GPs working in EDs.

### Sampling and recruitment of sites and participants

Ten case study sites were purposively selected and recruited for maximum variation (that is, model of GPED service); deprivation index; ED volume; and geographical location. Box 1 describes the different GPED models and GP remit at each case site. Models are adapted from a taxonomy described by Cooper *et al*.^6^ GPs working in GPED services either undertake a similar role to that in core general practice, or at some sites this is extended by ordering investigations beyond what is usually expected, or managing patients with increased acuity.

**Table:** How this fits in

Many people attending emergency departments (EDs) could be managed by GPs and employing GPs to work in or alongside emergency departments (GPED) is a way to address increased pressure on EDs. This study highlights the benefits of GPs choosing to work in GPED roles such as professional development, sharing skills and knowledge across both contexts, and retaining GPs in some capacity within the clinical workforce. However, GPs working in GPED services expressed concerns that the GPED model may not be sustainable and may also contribute to destabilisation of core general practice. By exploring the views, motivations, and experiences of GPs choosing to work in GPED roles, this study may inform planning and implementation of both GPED services and core general practice.

**Box 1.** GPED model by case site^a^

**Site**	**GPED model**	**GP role**
Birch	Inside ED: parallel	GP in ED (including investigations)

Chestnut	Inside ED: parallel	GP in ED (usual primary care)
	Outside ED: off-site	

Hawthorn	Inside ED: parallel (OOHs only)	GP in ED (usual primary care)

Juniper	Outside ED: on-site (OOHs only)	GPs either work in usual primary care role or adapt a dual role where they become involved in managing patients with ED health issues
	Inside ED: integrated	

Linden	Outside ED: hospital site + off-site	GP in ED (UCC) (usual primary care)

Nutmeg	Inside ED: parallel	GP in ED (UCC) (usual primary care)

Poplar	Outside ED: on-site	GP in ED (including investigations)

Redwood	Inside ED: parallel	GP in ED (including investigations)

Rowan	Inside ED: parallel	GP in ED (usual primary care)

Teak	Inside ED: parallel	GP in ED (including investigations,
	Outside ED: off-site	increased acuity)

a *Inside models — GPs co-located within ED. Can be: integrated — GPs work within the ED team managing a range of patients, or parallel — patients assessed as suitable for GP care ‘streamed’ to GP within the department. Outside models — patients assessed in ED then sent to GP outside ED, either within hospital grounds (on-site) or off hospital grounds (off-site). ED = emergency department. GPED = GPs working in or alongside EDs. OOH = GP out-of-hours service. UCC = urgent care centre.*

At one site (Juniper), GPs could work in a hybrid role across GPED services and ED managing a range of patient needs.

The qualitative research team approached GPs who worked within GPED sites to participate in interviews during case-site data collection. Although participants were recruited opportunistically, and through snowball sampling,^14^ the sample included a range of GPs in terms of experience (for example, from GP leads to newly qualified GPs) and their role/employment within and outside GPED services (Supplementary Table S1).

A total of 39 GPs were interviewed with 3 GPs interviewed twice during the study (42 interviews in total). Interviews took place across three timepoints (start/middle/end of study), and at sites where GPED services had been established for varying amounts of time, to attempt to capture the views of GPs who had experienced different forms of GPED over differing longitudinal periods.

### Data collection

Data were collected between October 2017 and December 2019. Interviews were semi-structured and primarily conducted face-to-face at case study sites, with a small number of interviews (approximately 10%) conducted by telephone at the participant’s request.

A topic guide was developed by the research team that was underpinned by the research literature and the wider General Practitioners and Emergency Departments Study’s research questions (Supplementary Box S1), and so was broader than is reported here. The topic guide allowed exploration of the professional background, motivations, views, and experiences of GPs working in GPED services reported in this paper. Participant information leaflets were provided to all participants and written consent obtained.

### Data management

Data management was compliant with the Data Protection Act (2018)^15^ and university data security policies. It was managed and held in accordance with General Data Protection Regulations (GDPR, https://gdpr.eu/).

Interview data were recorded and transported on an encrypted audio-recording device. All data were stored on the secure password-protected drive of a university server.

### Analysis

A broad coding framework was developed for the wider qualitative study by the research team to reflect the aims of the General Practitioners and Emergency Departments Study as a whole. Data were then summarised into case site pen portraits,^16^ compared/contrasted across sites, and thematically analysed.^17^ For the purposes of this paper, further, more nuanced, thematic analysis of GP interview data was conducted, on the broad theme of ‘perspectives of GPs in GPED’ — one of the overarching study aims. This involved one researcher re-analysing subthemes relating to GPs’ motivations, views, and experiences identified during the primary study analysis.

Findings were then discussed among the qualitative team. Pseudonyms were allocated to case sites (for example, Chestnut) and unique identifiers to individual participants.

## RESULTS

GPs worked in various roles in GPED services, across different GPED models. Some worked in GPED services as part of broader ‘portfolio working’, which describes GPs holding multiple roles. Four themes were generated that underpin the motivations, views, and experiences of GPs working in GPED services. The first two are concerned with the motivations of GPs working in GPED roles, and the latter two explore the experiences and views of GPs about the benefits and ramifications of utilising GPs in EDs for both core general practice and EDs.

### Motivation to work in GPED roles: the ‘pull’ of a portfolio a career — being a different kind of GP

Most GPs working in GPED roles enjoyed the challenge of working with more acutely unwell patients and saw it as a way of extending their scope of clinical practice, broadening their career, and offering potential new avenues for future working. This was particularly for newly qualified GPs who did not always consider core general practice their first-choice specialty, with GPED offering an opportunity to avoid being pigeon-holed within a linear career trajectory. These GPs positioned themselves as different from the majority of GPs who they considered more risk averse and less confident in their abilities to work in acute settings:

*‘There was another GP who worked* [in GPED] *on Mondays, but he didn’t feel confident to see some of the things they were sending us, so he doesn’t come any more … if you had people that are ready to see anything that comes in through the door then it will work really well. But then if people are happy to do that, they wouldn’t have become GPs in the first place.’* (Birch, GP.18)

GPs who chose to work in GPED services were moving away from traditional medical careers and forging new ways of working more centred on work–life balance and diversifying opportunities:

*‘Career wise, I think this job suits my work–life balance at the moment. I’m only doing this for two days which is five sessions … and the other days I’m able to work what I want to do in other places, locum or out-of-hours.’*

(Juniper, GP.62)

### Motivation to work in GPED: the ‘push’ of disillusionment with general practice

For some participants, working in GPED roles was seen less as a positive career choice or a genuine interest in the work; rather they worked there for pragmatic reasons. Some expressed disillusionment with core general practice, which was seen as highly pressurised and increasingly demanding compared with GPED. Some participants were reluctant to join GP partnerships that involved business, managerial, and employer responsibilities as well as increasingly complex clinical demands:

*‘Obviously, general practice in the community is really being hit hard. There are fewer GPs and quite a few of us are leaving partnerships for various reasons, so general practices are really under the cosh. With funding for district nursing, social services and all the things, it’s getting harder and harder, and we’re seeing more and more patients with more and more complex things.’*

(Rowan, GP.3)

In contrast to the open-ended commitments of core general practice, GPED involved one-off contacts with patients that require short-term decision making as opposed to long-term management of complex clinical issues. In particular, the pressure of time-limited consultations regularly experienced in core general practice was not encountered to the same degree in GPED:

*‘There is something refreshing about them not being your patients, dealing with them there and then, and then not having to deal with them thereafter. It makes it, in more challenging cases, less of a burden because, as a* [core] *GP, they’re always coming back to you, the ones you can’t do anything for. Whereas in ED it’s twenty minutes with them, or however long it takes. And then, in the nicest possible way, they’re someone else’s problem, they’re their own GP’s problem, they’re the ones with the responsibility for their ongoing care.’*

(Nutmeg, GP.19)

The flexibility of working in GPED services was considered by some to be more manageable in the long term. It was credited with extending medical careers that would previously have ended in early retirement or a move away from medicine because of burnout or ill health.

GPED offered an alternative way of working and this was reflected in the way GPs thought about and planned their careers:

*‘I came out of my partnership because of some health issues … I was really finding long, long days an issue. For me to come in and be able to practise the medicine I really enjoy, without actually having to do as many hours again doing paperwork, that has been great, and probably meant that I didn’t need to leave medicine.’*

(Rowan, GP.3)

### Views and experiences of GPED: professional reciprocity

Some interviewees saw working in GPED roles as a reciprocal learning opportunity. They viewed their GP expertise as a useful exchange for gaining and updating their own skills in emergency medicine, while bringing a general practice philosophy to EDs. They felt GPs initially presumed most patients were not seriously ill, but had the ability to identify sicker patients and escalate care when required.

Contrastingly, ED clinicians were considered to assume all patients were seriously ill until proven otherwise:

*‘To a GP a sore throat is a viral sore throat until you’ve got a real reason to suspect that they might have epiglottitis whereas to an ED doctor a sore throat could be epiglottitis until they’ve proved it’s not, so it’s a really different way of looking at things.’*

(Juniper, GP.39)

This philosophical divergence was thought to have practical consequences. ED clinicians were believed to order more investigations, admit more patients, and be less likely to take a ‘wait and review’ approach. Consequently, GPs felt their approach could be shared with secondary care clinicians for the benefit of patients. In return, GPs were able to update their skills in managing acutely ill patients, which would have the onward benefit of enhancing their core general practice work. GPs considered that working in GPED services enabled them to be role models for junior ED doctors and was mutually beneficial to both GPs and the wider healthcare system:

*‘Trying to cherry pick off the people that I know that I can see probably quite quickly and then get them moving, so either referred into the hospital or back home with GP follow-up or not. I think that works well and I think what some of the nursing staff quite often do* [is send them] *for an awful lot of blood tests that are not really necessary and actually, if they do come back abnormal, just confuse the picture and we end up hanging onto people who’re actually not really necessary.’*

(Juniper, GP.24)

GPs also valued the collaborative working of ED culture that was contrasted with feelings of isolation that sometimes occurred in core general practice.

Participants recognised the learning opportunities and informal support gained from other GPs working in GPED services and the wider ED. Working with other professional groups enabled GPs to enhance their skills, which benefited their core general practice:

*‘You feel like part of a team, and there’s that camaraderie which is quite nice. It’s nice to have the group of GPs and get their perspective on general practice in a setting like this. We’re working alongside experienced nurse practitioners, asking them for advice on things like musculoskeletal things and that will actually go to aiding me when I’m in the community.’*

(Teak, GP.6)

GPs working in GPED roles also felt they could facilitate collegiality between primary and secondary care. Their experience of core general practice allowed them to challenge the flaws in systems for the benefit of patients and in support of core general practice:

*‘… our other role I think is to improve things for primary care so that actually we don’t get inappropriate things being asked of primary care from secondary care. One of the things that I’ve done is introduced sick note certifications … we should not be sending them back to general practice just to get a sick note … So, I think GPs have a role of being here and standing up for general practice.’*

(Juniper, GP.24)

### Views and experiences of GPs: sustainability of GPED services and core general practice

Participants identified several challenges to the sustainability of GPED services. It was perceived that some doctors were attracted to general practice to avoid working shifts, weekends, and evenings. In this way, these GPs saw themselves as different from the norm. Consequently, there was concern that the unsociable hours of GPED would reduce the pool of GPs interested in this type of work. Lack of GPs with the desire or requisite skills to work in GPED roles meant services were not always fully staffed, reducing the perceived impact of GPED services:

*‘I’m not sure we’re going to suddenly stumble across a large cohort of GPs who are particularly well trained in minor injuries, number one. And the other thing we haven’t really discussed is often part of their reason for becoming GPs is a decent lifestyle and decent hours. Coming to now spend Friday night, Saturday night dealing with drunks who’ve punched each other is not such an attractive prospect.’*

(Chestnut, GP.24)

Remuneration was important in choosing to work in GPED roles. However, views and experiences differed. Some found working in GPED services was competitively rewarded whereas others were better remunerated elsewhere. Competition for GPs’ skills and expertise between services, such as GP out-of-hours services and urgent care centres, led in some places to a deficit of suitably qualified GPs willing to work in GPED services, which caused pay inflation.

However, where GPED services could not afford competitive pay, they either lost out and were under-resourced, or different strategies for attracting GPs were developed:

*‘there needs to be a real incentive and either it needs to be we’re offering something exciting and different and interesting, so you can be part of a new, exciting team … or we’re offering a financial incentive and even though we’re offering a consultant-level salary scale that still is difficult to compete with GP out-of-hours … one of our applicants does … out-of-hours and we can’t compete with it. So, he’s pulled out.’*

(Juniper, GP.39)

Despite these concerns, by enabling GPs to develop skills and broaden their scope of practice, GPED was considered to have the potential to retain GPs in some form of general practice for at least some of the time. Development of novel and portfolio roles were thought to reduce burnout from working in one specialty, whether ED or general practice. In addition, exposing junior doctors to GPs’ work in ED and portfolio working was considered to encourage them to consider various forms of general practice as attractive career options, which may consequently boost the number of future GPs. However, it was anticipated that, to be sustainable, initiatives to develop novel general practice roles require support from the broader medical training system and relevant royal colleges:

*‘The combination* [of ED and GP] *I think actually it’s not for everyone but it’s a really attractive career option, so I think it may improve recruitment into both subjects, ED and GP, and it’d be nice to see the College back that up, maybe try and develop something like* [an] *interface medicine diploma or more qualifications.’*

(Juniper. GP.24)

GPs are a finite resource and competing services recruiting from the same pool of GPs was a concern to participants. They felt that instead of GPED reducing pressure on general practice, it added burden by diverting qualified staff from an already under-resourced and pressurised core general practice service. Increasing workload pressures and limited funding within core general practice, along with favourable GPED conditions and pay, meant GPED was seen as a more attractive workplace. These pressures had the potential to be cyclically exacerbated as fewer GPs working in core general practice would potentially lead to fewer general practice appointments, increasing the burden on wider general practice and the volume of patients attending ED with primary care problems:

*‘I think it is getting harder to recruit GPs into general practice. I think there are four or five competing services. There are only ever going to be a finite number of GPs.’*

(Rowan, GP.10)

Despite working in or alongside EDs, some participants thought GPs should not contribute to the ongoing depletion of core general practice but focus instead on where GP resources are needed most. Several GPs felt ambivalent about, or did not agree with, GPED as a policy and considered it contributed to further system complexity and duplication. Ultimately, some GPs felt GPED and similar initiatives destabilised core general practice and proposed a whole-system approach to address restructuring and funding, rather than piecemeal initiatives that were considered to have limited effects:

*‘The way our model has been set up, I think what will be best for the patient is going to, potentially, be destabilising for the general practice community … Really, we need to look at the whole system in terms of that. I think without that, the thing is just going to fall apart, isn’t it? You’re taking out a matrix and just leaving the rungs.’*

(Teak, GP.26)

## DISCUSSION

### Summary

This study provides insight into the views, motivations, and experiences of GPs working in GPED services across England to inform the planning and implementation of these services alongside wider general practice provision. GPs in this study worked within a variety of GPED models and, because the models were so diverse, it was not possible to draw specific conclusions about how this may have affected the perceptions and views of GPs. It does, however, highlight the complexity involved in delivering GP services in EDs, as well as indicating the variety of different experiences of GPs working in GPED roles.

Established GPs in this study used GPED roles to maintain a medical career when they perceived core general practice to be unmanageable. For less experienced GPs, GPED roles provided a means of engaging with medicine in ways that fit more closely with career and life aspirations. As such, GPED can be argued to offer an important way of keeping the skills and knowledge of experienced GPs, while ‘growing’ future GPs. Lessons learned from GPED could potentially be used in similar initiatives elsewhere.

As well as perceived support for new, flexible ways of working, GPED was considered to facilitate an environment in which GPs were in demand with subsequent remuneration and wider benefits. GPED promoted general practice to the hospital workforce and provided GPs with enhanced skills transferable to their core general practice work. However, a number of unintended consequences of GPED services were identified, including the possible destabilisation of core general practice though shifts in funding and ‘poaching’ of an already depleted GP workforce. The sustainability of GPED itself was questioned as services competed for finite GP resource.

Although GPED roles were considered a positive career opportunity for this self-selecting group of clinicians, there was scepticism as to whether this was applicable to the wider GP population whose work choices and requirements may differ.

### Strengths and limitations

This study reports findings from an opportunistic sample of GPs working in or alongside EDs at 10 case sites across England. The GPs interviewed had chosen to work in GPED roles and the ways in which GPED services functioned between case study sites varied significantly. Consequently, findings cannot necessarily be considered representative of GPs as a professional group or across different models of GPED, and in particular will not capture the views of GPs who might have considered working in GPED roles under different circumstances. Similarly, in such a rapidly changing environment, studies such as this one provide a cross-sectional snapshot of current issues that may change over time and, as such, should be viewed within this context.

However, there are commonalities and consistencies across contexts, and findings align with and extend national and international literature.^8^^,^^11^^,^^12^^,^^18^ The study highlights issues that may resonate with primary care clinicians more broadly, as well as indicating a range of issues relating to both GPED and wider general practice that require further consideration and exploration.

### Comparison with existing literature

GPs were attracted to GPED roles as it offered flexible ways of organising work and enabled them to develop portfolio careers. Portfolio working is increasingly popular among medical practitioners, particularly those who are younger and more recently qualified.^18^ GPs with portfolio careers are less likely to consider leaving practice than those who work exclusively in core general practice.^9^ However, lack of formal professional support for hybrid and portfolio roles was identified in the current study and is reflected elsewhere in the literature.^18^

In the current study, GPs valued the collaborative working of GPED. The value of shared learning between GPs and ED clinicians has been highlighted elsewhere.^8^ Job satisfaction improves when workload is shared within a team,^19^ and integration between primary and secondary care has been found to improve patient care.^20^ Consequently, team working as exemplified in GPED may improve the experiences of both clinicians and patients.

Pressures of core general practice are leading GPs to consider alternative career options.^9^^,^^19^^,^^21^ In the current study, less experienced GPs lacked interest in traditional general practice partnerships and did not plan their career at an early stage, which is consistent with previous findings.^12^^,^^21^ Disillusionment with core general practice was found in the current study and this provided significant impetus to drive GPs towards GPED roles, and these findings extend previous research by highlighting that by providing room for GPs to focus on discrete episodes of clinical work, and workforce flexibility, GPED roles may provide a suitable alternative or adjunct to core general practice for at least a subset of GPs.

However, there was scepticism among participants that a critical mass of GPs would be willing to staff GPED services as working patterns and conditions were considered inconsistent with what most GPs want. A recent study of GPED services identified difficulties in filling rota gaps and unsociable shift patterns,^8^ although the reasons for this were unclear. Previous studies have identified that medical practitioners choose to work in core general practice as they perceive it to lack unsociable hours while providing work–life balance and continuity of care.^11^^,^^12^ This may be a limiting factor for GPs choosing to work in GPED services.

In the current study, there were contradictions between competing assertions that GPs were attracted to GPED roles because it offered work–life balance in terms of limited commitment and defined, time-limited shifts, and that most GPs did not want to work unsociable hours. It is likely that different GPs have a variety of requirements. Participants in the current study credited GPED services with supporting core general practice by retaining GPs in some form of general practice, for at least some of the time, and promoting general practice to junior doctors. New approaches to GP careers are required to retain GPs^21^ and the findings in the current study suggest GPED may offer one of a range of approaches to address this.

Of significance is that participants in this study considered that system-wide change was necessary to address pressures on both core general practice and EDs, rather than adopting a single-initiative approach. This resonates with views of GPs working in core primary care who argue that a multifactorial approach is required to achieve effective and sustained solutions to workforce issues among GPs.^21^

### Implications for research and practice

This study has a number of implications for practice. Box 2 outlines considerations that may enable GPED to develop to support both GPED services and core general practice. This may be used when planning and implementing GPED policy and services to work effectively alongside wider general practice provision.

**Box 2.** Suggestions for future practice

**Sustainability of GPED**	**Sustainability of core general practice**	**Sustainability of both GPED and core general practice**
Offer flexibility in work patterns, for example, shift patterns, locum, and part-time work balanced with need for effective service planning and provision	Systemic change required to make general practice sustainable, for example, flexible working, collaborative working environment	Support for portfolio careers such as working in GPED roles may allow some GPs to extend their working life, reduce early retirement and attrition, and prevent burnout
Support GPs to broaden clinical opportunities when working in GPED services to enhance the core GP role	Support flexibility of workload organisation, for example, consultation duration	Portfolio working and novel GPED roles require structured education and support from training systems and medical royal colleges
Support for GPs to adapt to different ways of working in GPED roles according to their career plan and aspirations	Use GPED to promote general practice to junior doctors and other healthcare professionals working in ED	Consideration of strategies to prevent/limit competition for finite GP resource
Development of strategies to reduce competition for GP resource	Potential for core general practice to ‘borrow’ learning from the positive benefits GPs experience in GPED roles	Develop strategies that value shared learning and support for collaborative working
Provide ‘incentives’ to work in GPED services such as support and supervision, career planning	Supporting GPs to work part-time in GPED services or other portfolio working, as well as working part-time in core general practice, may assist retention of GPs	Develop the GPED role to include input into system changes, for example, streamlining primary/secondary care referral processes
Utilise GPs as a resource to share learning and primary care philosophies with ED colleagues	GPED roles can be used as a ‘stepping stone’ for those GPs yet to make longer-term career decisions	Professional reciprocity and broadening clinical opportunities enhances both primary and secondary care

*ED = emergency department. GPED = GPs working in or alongside EDs.*

GPs in this study found working in GPED services to be personally and professionally beneficial. It suited their interests and allowed working and lifestyle flexibility not usually afforded in core general practice. However, the attraction of GPED was to a large extent driven by disillusionment with core general practice. This means that, unless underlying issues are addressed, GPED has the potential to further destabilise general practice, with the negative consequence of creating increased pressure in both core general practice and EDs.

This study provides a number of considerations for both the development of future GPED services and for core general practice, such as the positive effects of professional reciprocity, collaboration, and personal development. GPED may offer an opportunity to support and extend clinical practice for more experienced GPs, while providing mechanisms for recruiting and retaining younger and more recently qualified GPs. However, while such initiatives are developed in an *ad hoc* way and without supporting system-wide changes, the potential of GPED may be limited and further depletion of the core general practice workforce a continuing area of concern.
